# On Empyroform, a Dry and Almost Odorless Tar Preparation

**Published:** 1903-10

**Authors:** 


### On Empyroform, a Dry and Almost Odorless Tar Preparation.

Dr. Bruno Sklarek, from Professor Neisser's Dermatological Clinic of Breslau University, (*Therapie der Gegenwart,* July, 1903,) says: practitioners are well aware of the fact that tar, besides its efficacy in psoriasis, the various forms of lichen and the pruriginous dermatoses in general, is the most valuable remedy that we possess for the treatment of eczema. Yet its proper employment is often a matter of difficulty, for the drug is hard to handle and may do harm as well as good.

Subsidiary but also important difficulties in the employment of tar are its black color and an odor which is very objectionable to many persons.

These considerations have for years led to persistent attempts to find a drug to take its place. My own efforts have been directed towards the employment of a condensation product of tar and formaldehyde, prepared by the Schering chemical factory of Berlin, and put upon the market under the name of empyroform.

My efforts were directed to ascertain the relationship of empyroform to tar in its therapeutic action, and to finding out whether it had similar properties to the older preparation or advantages over it.

Empyroform is a dry, non-hygroscopic, brownish powder, with a peculiar weak odor in no way resembling that of tar. It readily gives off formaldehyde when heated. It is insoluble in water, but dissolves in aceton and the caustic alkalies, and still more readily in chloroform. Its color and weak odor give it some prima facie advantages over tar. An empyroform-zinc paste is grey, whilst a tar-zinc paste of equal strength is black. The absence of marked odor rendered the preparation especially acceptable to all the patients.

In powder form, either pure or mixed with zinc and amylum, empyroform was used almost exclusively in moist eczemas; and of course, like every other powder, only in conjunction with a salve-muslin, to prevent injury to the skin when renewing the application. We found the dressing very useful in these cases; but a mixture of the empyroform in a salve or a zinc paste was generally more convenient and efficacious.

In consequence of its desiccating properties empyroform is very useful in suspension, and can be added in varying amounts to the base mixture usually employed for that purpose (zinc, oxyd., talc. venet., glycerin., aq. dest., ana p.e.). It desiccates very rapidly, however, and should not be prescribed in too large amounts at one time. A good formula is the following:

℞ Empyroform....................................15.0 (½ ounce.)
Talc. venet................................
Glycerin ..................................ana 10.0 (2½ drams)-
Aq. dest. ....................................20.0 (5 drams-)
or instead of the last
Spirit. vini and aq. dest ...................ana 10.0 (2½ drams).
M Paint. To be well shaken before applying.

These suspensions have proved very valuable, the patients liking them better than the ointment. They are especially appropriate for individuals with an idiosyncrasy for fats. As with all similar applications, they are useful only when there is not much exudation present, either in the early erythematous stages of eczema, or later when desiccation and scaling have already set in.

The new remedy can be used in the form of tincture or varnish very advantageously; its color is dark then, but it is almost odorless. I found that a simple solution in chloroform in the proportion of 1 to 3 was too brittle and did not adhere sufficiently well to the skin. I therefore used the following tincture, which has not this disadvantage:

Empyroform...............5.0-10.0 (1¼ drams to 2½ drams).

Chloroform................

Tinct. Benz............ana ad, 50.0 (1½ ounces).

M. Sig. Paint.

Finally, one especial advantage of empyroform remains to be noted. It can render individuals who cannot stand tar at all, capable of using the drug. The ideal eczema treatment is the tar treatment. It is a very great advantage to have a remedy in empyroform to help us even in cases that are most recalcitrant and susceptible to the tar. I have used it repeatedly in cases of the kind and have only good results to report. Patients who could not stand tar in any form, in whom its most cautious application caused irritation and new attacks of the eczematous disease, have become so accustomed to the drug through the empyroform treatment that they were finally not only able to use a weak tar-zinc paste, but even stood the tar tincture without any trouble, and so were finally led to a definite cure.

The advantages of the new drug are probably due to the combination of formaldehyde with the tar. To recapitulate them, the first are its great antipruritic and desiccating qualities. Then it causes neither local reaction nor systemic intoxication. Further, with its help patients can be gradually accustomed to the use of tar. It is almost entirely odorless. The lesser intensity of its color is a property whose value must not be underestimated; it is a more cleanly dressing, and does not soil the body and bed linen as tar does. All these things give it the right of introduction into the field of dermatotherapy.

